# Antibodies Against Angiotensin II Receptor Type 1 and Endothelin A Receptor Are Associated With an Unfavorable COVID19 Disease Course

**DOI:** 10.3389/fimmu.2021.684142

**Published:** 2021-05-13

**Authors:** Jelle Miedema, Marco Schreurs, Simone van der Sar – van der Brugge, Marthe Paats, Sara Baart, Marleen Bakker, Rogier Hoek, Willem Arnout Dik, Henrik Endeman, Vincent Van Der Velden, Adriaan van Gammeren, Antonius Ermens, Joachim G. Aerts, Jan Von Der Thüsen

**Affiliations:** ^1^ Department of Pulmonology, Erasmus Medical Center, Rotterdam, Netherlands; ^2^ Department of Immunology, Erasmus Medical Center, Rotterdam, Netherlands; ^3^ Department of Pulmonology, Amphia Hospital, Breda, Netherlands; ^4^ Department of Biostatistics, Erasmus Medical Center, Rotterdam, Netherlands; ^5^ Department of Intensive Care Medicine, Erasmus Medical Center, Rotterdam, Netherlands; ^6^ Department of Clinical Chemistry and Hematology, Amphia Hospital, Breda, Netherlands; ^7^ Department of Pathology, Erasmus Medical Center, Rotterdam, Netherlands

**Keywords:** COVID19, Endothelin Receptor Type A Antibody, Angiotensin II Receptor type 1 Antibody, Antinuclear antibody (ANA), Autoimmunity, Prognosis (D011379)

## Abstract

**Background:**

Lung histopathology demonstrates vasculopathy in a subset of deceased COVID19 patients, which resembles histopathology observed in antibody-mediated lung transplant rejection. Autoantibodies against angiotensin II type 1 receptor (AT1R) and Endothelin receptor Type A (ETAR) have been demonstrated in antibody-mediated rejection and may also be associated with severe COVID19 infection. Objective To assess AT1R and ETAR auto-antibodies in COVID19 patients and controls, and explore their association with disease course.

**Methods:**

65 hospitalized patients with COVID19 infection were included. Clinical and laboratory findings were retrospectively assessed. Patients with unfavorable disease course, admitted at the intensive care unit and/or deceased during hospital admission (n=33) were compared to admitted COVID19 patients with favorable disease course (n=32). The presence of antinuclear antibodies (ANA) and auto-antibodies against AT1R or ETAR in peripheral blood were compared between COVID19 with unfavorable and favorable disease course and age matched controls (n=20).

**Results:**

The presence of ANA was not significantly different between COVID19 patients with unfavorable (n=7/33; 21%) and favorable disease course (n=6/32; 19%) (p= 0.804) and controls (n=3/20; 15%). Auto-antibodies against AT1R were significantly increased in unfavorable disease course (median 14.59 U/mL, IQR 11.28 – 19.89) compared to favorable disease course (median 10.67 U/mL, IQR 8.55 – 13.0, p< 0.01). ETAR antibody titers were also significantly increased in unfavorable disease course (median 7.21, IQR 5.0 – 10.45) as compared to favorable disease course (median 4.0, IQR 3.0 – 6.0, p <0.05).

**Conclusion:**

Auto-antibodies against AT1R and ETAR are significantly increased in COVID19 patients with an unfavorable disease course.

## Introduction

The disease course of infection with Severe Acute Respiratory Syndrome Coronavirus 2 (COVID19) is highly variable, from asymptomatic to pneumonia and acute respiratory distress syndrome with multiorgan failure (1). Lung histopathology demonstrates diffuse alveolar damage with vasculopathy, angiocentric inflammation and microthrombi in a subset of deceased COVID19 patients (2). Increasing evidence shows that endothelial dysfunction plays an important role in severe COVID19 disease progression and associated coagulopathy (2, 3). The observed pulmonary vascular damage in COVID19 patients resembles antibody-mediated rejection (AMR) after lung transplantation. Although AMR is usually caused by the formation of antibodies directed at the donor specific human leucocyte antigen (HLA) system, it has also been associated with non-HLA auto-antibodies against the G-protein coupled receptors Angiotensin II Receptor type 1 (AT1R) and Endothelin receptor Type A (ETAR) (4, 5). The similarity between vasculopathy in severe COVID19 and AMR after lung transplantation raised the question whether similar autoimmune mechanisms contribute to COVID19 pathogenesis.

## Method

We assessed AT1R, ETAR and antinuclear antibodies (ANA) in peripheral blood of n=65 COVID19 patients, admitted in March and April 2020 to the Erasmus University Medical Center (n=21) and Amphia Hospital (n=44) and n=20 age matched elderly controls. Infection with SARS CoV2 was confirmed with polymerase chain reaction in all patients. Clinical and laboratory findings were retrospectively assessed. We defined the COVID19 disease course as unfavorable if patients were admitted to the intensive care unit (ICU) and/or died during hospital admission (n=33) and favorable in patients treated on the general ward (n=32). Because of the non-interventional design, this study was exempt from ethics approval by the Institutional Review Board of Erasmus MC and Amphia Hospital. Laboratory measurements for the presence of AT1R and ETAR antibodies were performed on available blood samples from COVID19 patients at the Laboratory Medical Immunology of the Erasmus MC (Rotterdam, the Netherlands). Anti-AT1R and –ETAR were determined in serum (obtained by venepuncture followed by 30 min. clotting of peripheral blood and 5 min. centrifugation at 3000 g) using a CE-marked enzyme immuno-assay (EIA), developed at CellTrend GmbH (https://www.celltrend.de/en/elisa/in-vitro-diagnostika-human/), according to the manufacturer’s instructions. Available blood samples were collected 6.4 days after admission in the favorable group, compared with 8.6 days in the unfavorable group (p = 0.037, **Table 1A**). For both antibodies against AT1R and ETAR the same concentration of 10 U/mL was considered the cut-off value (10-17 U/ml borderline and >17 U/ml positive) in line with the manufacturer’s recommendation based on previous validation (6). Anti-nuclear antibodies (ANA) were determined using classical indirect immunofluorescence on HEp2 cells, at 1:80 serum dilution, according to standard protocol. Only nuclear immunofluorescence patterns were considered ANA positive. Differences in clinical and laboratory characteristics were assessed between the control and overall COVID group and between the two outcomes in the COVID group. Continuous outcomes were analyzed with independent samples t-tests or Mann-Whitney U tests in case of non-normality and categorical outcomes with Chi-squared or Fishers exact tests, where appropriate. Repeated AT1R sample were analyzed with a Wilcoxon signed-rank test. Differences between the three groups in AT1R and ETAR titer were calculated using independent samples Kruskal-Wallis tests. Post hoc comparisons between two specific groups were analyzed with Mann-Whitney U tests. The Holm method was used to correct for multiple testing. For categorical ANA testing, chi-square testing was used. *P* values < 0.05 were considered significant.

**Table 1A:** Demographics and Clinical Features of COVID19 Patients Classified as “favorable” and “unfavorable” disease course.

	Controls (n = 20)	All COVID19 patients (n = 65)	P-value*	Favorable disease course (n = 32)	Unfavorable disease course (n = 33)	P-value**
Male sex – n (%)	10 (50)	49 (75)	0.050	21 (66)	28 (85)	0.072
Age - yr (mean- range)	59.8 (56-64)	66.9 (25-87)	<0.0001	69.7 (49-87)	66.0 (25-87)	0.067
Body mass index (BMI) (mean-range)	Unknown	28.1 (19-42)		27.8 (22-39)	28.4 (19 – 42)	0.632
History of smoking – n (%)	Unknown	Current 0 (0) Former 18 (28) Never 21 (32) Unknown 26 (40)		Current 0 (0) Former 9 (28) Never 15 (47) Unknown 8 (25)	Current 0 (0) Former 9 (27) Never 6 (18) Unknown 18 (55)	0.170
Comorbidity: Diabetes – n (%)	2 (10)	16 (25)	–	8 (25)	8 (24)	0.943
Comorbidity: cardiovascular n (%)	0 (0)	20 (31)	–	10 (31)	10 (30)	0.934
Comorbidity: hypertension n (%)	1 (5)	40 (62)	–	21 (66)	19 (58)	0.505
Comorbidity: renal n (%)	0 (0)	3 (5)	–	1 (3)	2 (6)	–
ACE or AT2 inhibitor - n (%)	–	18 (28)	–	8 (25)	10 (33)	0.633
Mean days between first symptoms and admission (range)	–	8.8 (1-19)	–	9.0 (1-14)	8.5 (3-19)	0.649
Mean days between admission and sampling of antibodies (range)	–	7.5 (1-21)	–	6.4 (1-13)	8.6 (2-21)	0.037
Anti-nuclear antibody (ANA) – n (%)	3 (15)	13 (20)	0.752	6 (19)	7 (21)	0.804
CRP mg/L (median- IQR)	–	82 (27-204)	–	38 (23-91)	170 (72-304)	<0.0001
Steroids during admission - n (%)	–	11 (17)	–	5 (16)	6 (18)	0.783
Remdesivir during admission - n (%)	–	0 (0)	–	0 (0)	0 (0)	–
Chloroquine during admission - n (%)	–	35 (54)	–	26 (81)	9 (27)	<0.0001
ICU admission - n (%)	–	29 (45)	–	0 (0)	29 (88)	–
Supplementary oxygen	–	65 (100)	–	32 (100)	33 (100)	–
Invasive ventilation - n (%)	–	229 (45)	–	0 (0)	229 (88)	–
Mortality – n (%)	–	12 (18)	–	0 (0)	12 (36)	–

*P-value comparing healthy controls with all COVID19 patients, **P-value comparing Covid patients with favorable and unfavorable outcome. No statistical inferences were made in groups with <3 cases.

## Results

The mean time between the start of COVID19 symptoms and hospital admission was 8,8 days and did not differ between patients groups (p = 0.67, **Table 1A**). During admission, n=11/65 (17%) patients were treated with corticosteroids.

**Figure 1:**
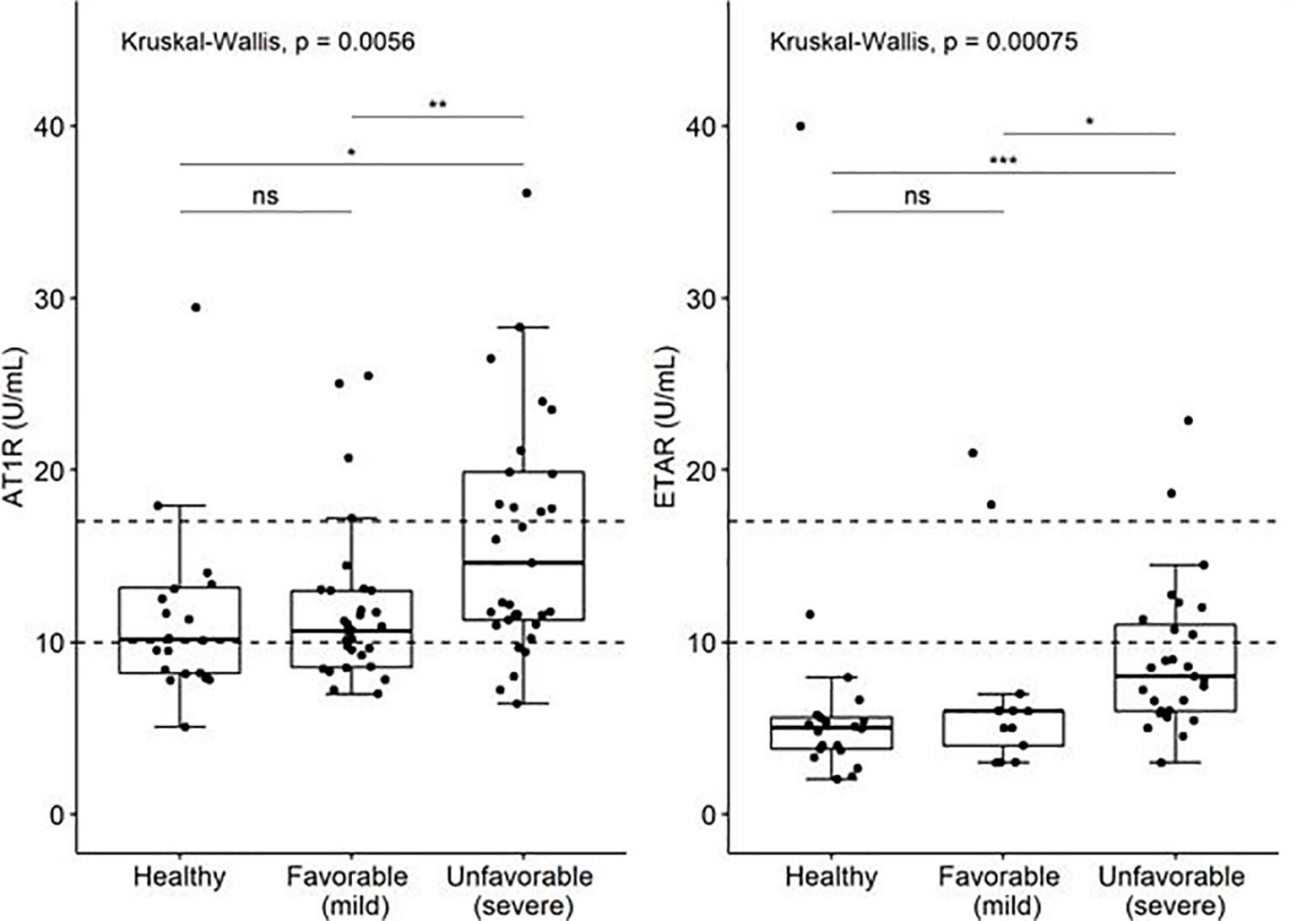
Levels of auto-antibodies against AT1R and ETAR (U/ml) in COVID19 patient with favorable and unfavorable disease course and age matched controls. The limits of the axes in the graph are placed at 45 U/ml. Three values of AT1R are outside the range of the graph; they are included in the analysis. P-values: ns, not significant (>0.05), "*" <0.05, "**" <0.01, "***"<0.001.

Viral infections can trigger elevated titers of autoantibodies during inflammation (7). Therefore, we determined antinuclear antibody (ANA) presence in peripheral blood of COVID19 patients. No significant differences were observed in ANA presence between total COVID19 patients and controls, or between COVID19 patients with favorable (n=6/32; 19%) and unfavorable disease course (n=7/33; 21%) (p= 0.804; **Table 1A**). Subsequently, autoantibodies against AT1R and ETAR in patients and controls were evaluated. AT1R antibody titers were significantly increased in an unfavorable disease course (median of 14.59 U/mL, IQR 11.28 – 19.89) compared to a favorable disease course (median 10.67 U/mL, IQR 8.55 – 13.0, P< 0.01) (**Table 1B**, **Figure 1**). Positive AT1R titers (> 17 U/ml) were found in n=14/33 (42%) patients with an unfavorable disease course compared to n=3/32 (9%) patients with a favorable disease course or n= 3/20 (15%) controls. ETAR antibody titers were also increased in an unfavorable disease course (median 7.21 U/mL, IQR 5.0 – 10.45) compared to a favorable disease course (median 4.0 U/mL, IQR 3.0 – 6.0), but the number of positive titers (>17 U/ml, 2/33 (6%) for unfavorable disease course compared to 2/32 (6%) for favorable disease course) did not differ between groups (**Figure 1**). Within the unfavorable disease group, n=12 patients died during hospital admission. The median AT1R and ETAR antibody titers in the deceased patients were 11.48 U/mL (IQR 9.56-21.95) and 5.34 U/mL (IQR 4.16-8.47) respectively. In the unfavorable disease group, deceased patients did not have significantly different AT1R and ETAR antibody titers compared to the patients on the ICU that survived (p = 0.225 and p = 0.082 respectively).

**Table 1B:** Titers of AT1R and ETAR in subgroups, with median and interquartile range (IQR).

Variable	Controls(n = 20)	Favorable disease course (n = 32)	Unfavorable disease course (n = 33)	P-value**
AT1R (U/mL) Median (IQR)	10.15 (8.19 – 13.14)	10.67 (8.55 – 13.0)	14.59 (11.28 – 19.89)	<0.01
ETAR (U/mL)	5.04 (3.79 – 5.65)	4.0 (3.0 – 6.0)	7.21 (5.0 – 10.45)	<0.05

**P-value comparing COVID19 patients with favorable and unfavorable outcome. For comparison between control and disease groups, see **Figure 1**.

In 8 patients with a high AT1R serum titer serum samples of a previous blood sampling were available. To investigate the AT1R titer over time, we analyzed the earlier samples. In this small cohort, n=6/8 patients had increasing serum AT1R titer over time and n=4 patients with increasing AT1R titer had unfavorable disease course. The median AT1R titer was 13.5 U/ml (IQR 11.0-21.5) at the earlier time point, compared to 21.0 U/ml (IQR 18.5-24.5) in the original samples from the cohort (p = 0.103).

## Discussion

Increased autoantibodies have been demonstrated in COVID19 patients (8, 9). Recently, neutralizing IgG antibodies against several types of interferon were found in life-threatening COVID19, while no such autoantibodies were found in individuals with mild disease (10). These findings suggest autoantibodies could be an important part of the immunological puzzle of clinical variability in COVID19.

Our study is the first report on increased autoantibodies against AT1R and ETAR in COVID19 patients. Antibodies against AT1R bind to the angiotensin II Type 1 receptor, which regulates water-salt balance and blood pressure. They have previously been found to be able to activate the AT1R, mimicking the proinflammatory effect of angiotensin II (5). Anti-ETAR antibodies exhibit chemotactic activity and stimulate neutrophil trafficking (11). Both AT1R and ETAR antibodies have been linked to cardiovascular disease, hypertension and aging (12) and may contribute to acute and chronic AMR in transplant patients (4, 13). We found increased titers of AT1R and ETAR antibodies in severe COVID19 compared to patients with favorable disease course, while ANA titers did not differ between groups. Autoantibodies can transiently increase during critical illness secondary to immune activation (14). A nonspecific increase in autoantibodies secondary to virus-induced immunostimulation cannot be excluded, but increased autoantibodies against AT1R and ETAR in severe disease with equal distribution of ANA titer between groups is supportive of a specific autoimmune response in the current study.

The cause of death in n=11/12 patients was non-resolving ARDS with associated complications (e.g. multiple organ failure or pulmonary fungal infection), directly related to COVID19 infection. One COVID19 patient died of complications related to a bone fracture and delirium. This patient had low AT1R and ETAR antibody titers. Treatment with corticosteroids did not differ between groups and was only given to 17% of the patients, as this was not standard care during the first months of the pandemic. Treatment with chloroquine was higher in the favorable group, because these patients were only included from Amphia Hospital where this was the local treatment protocol. Samples from patients with an unfavorable disease course were included from both hospitals. Mechanical ventilation was (by definition) only applied to patients on ICU with an unfavorable disease course and an influence on AT1R and ETAR antibody formation could not be ruled out.

We hypothesize that an increase in AT1R and ETAR antibodies may be a consequence of severe vascular endothelial damage in a proinflammatory environment. An alternative explanation could be that AT1R and ETAR antibodies are increased in COVID19 high risk groups with associated cardiovascular disease, hypertension and increased age. Although the clinical significance of AT1R and ETAR antibodies in severe COVID19 remains unknown, the current finding is intriguing. These antibodies have been demonstrated to affect several biological mechanisms (5, 11) and more research is needed to determine if increased AT1R and ETAR autoantibodies may aggravate -or even control - tissue injury. In view of the central role of ACE-2 receptor in cellular viral entry of SARS-CoV-2, in combination with the importance of the renin-angiotensin-aldosterone-system (RAAS) in maintaining endothelial homeostasis, a putative effect of inhibition of ACE and AT2 on COVID-19 severity has been proposed (15–17). In the initial phase of the disease, ACE-2 could be targeted to hamper SARS-CoV-2 replication. Thus, ACE inhibitors (ACEIs) could prove advantageous in the prevention of severe COVID-19 pneumonia. Conversely, especially in later stages of the disease, a virus-induced reduction in ACE-2 may also lead to an increase in angiotensin-II levels, which could have direct deleterious effects through activation of AT1R, in some patients compounded by agonistic anti-AT1R antibodies. Timing of ACE inhibitor therapy may therefore have significant impact on its net beneficial or deleterious effect. In our cohort, the use of ACE or AT2 inhibitors did not differ between favorable and unfavorable groups (P value 0.63). Unfortunately, the groups were too small and the data regarding individual clinical functional parameters insufficiently detailed to allow for in-group comparisons. Inhibition of AT1R signaling with AT1R blockers (ARBs) such as telmisartan and losartan to reduce the harmful effects of Ang II/AT1R axis while enhancing the ACE2/Ang (1–7) protective axis, has also been proposed as a strategy to counteract its excessive stimulation in COVID-19, with resulting microvascular inflammation, coagulation and fibrosis. Similarly, the agonistic effect of anti-ETAR1 antibodies could potentially be mitigated by the use of endothelin receptor agonists such as the ETAR-selective antagonist ambrisentan or the dual ETAR and ETBR antagonist bosentan (12). In a series of recent studies, however, the use of ACEI and ARBs has, on balance, not been found to be unequivocally associated with severity of disease (18). This type of observational research is hampered by confounding factors and bias, however, and whether any of these treatments have a positive effect on the outcome of COVID-19 pneumonia remains to be definitively determined and requires further study.

Outside possible pathophysiological effects of AT1R and ETAR antibodies, the increase in severe disease is interesting as they might serve as clinical biomarkers for severe vascular damage in COVID19 patients. Taking into account the cut-off value for positive results, an increase in AT1R antibody seems clinically relevant and further study is warranted.

Our study has some limitations. Firstly, samples from patients with a favorable disease course were included from Amphia Hospital, while samples from patients with an unfavorable disease course were included from both hospitals. Secondly, available serum samples were taken at a variable time point during the disease course. We were not able to retrospectively correlate the antibody titers to multiple inflammatory markers from the same time point such as d-dimer and interleukins, or determine the presence of antibodies at first presentation. Future prospective research is therefore necessary to determine whether these antibodies have a role as biomarker or have a role in pathogenesis of vascular damage in COVID19.

In conclusion, our study demonstrates increased levels of AT1R and ETAR autoantibodies in COVID19 patients who were admitted to intensive care and/or died during hospital stay, compared to patients with a favorable COVID19 outcome.

## Data Availability Statement

The original contributions presented in the study are included in the article/supplementary material. Further inquiries can be directed to the corresponding author.

## Ethics Statement

The studies involving human participants were reviewed and approved by The Medical Ethics Committee Erasmus MC of Rotterdam, The Netherlands. Written informed consent for participation was not required for this study in accordance with the national legislation and the institutional requirements.

## Author Contributions

JM, MS, SmS, MP, SB, RH, MB, JA, and JvT made substantial contributions to the conception and acquisition, drafting, analysis and interpretation of data for the work. All listed authors helped acquire data, revising it critically for important intellectual content, analysis and interpretation. All authors are in agreement to be accountable for all aspects of the work in ensuring that questions related to the accuracy or integrity of any part of the work are appropriately investigated and resolved. All authors contributed to the article and approved the submitted version.

## Funding

Department of pulmonary medicine, Erasmus Medical Center, Rotterdam & ZonMW funding, Project number 10430012010016.

## Conflict of Interest

JM reports personal fees from Boehringer Ingelheim, Roche and Chiesi, all outside the submitted work. SS-B reports personal fees from Astra Zeneca, Chiesi, ALK, Novartis, GSK, all outside the submitted work. JA reports personal fees and non-financial support from MSD, personal fees from BMS, Boehringer Ingelheim, Amphera, Eli-Lilly, Takeda, Bayer, Roche, Astra Zeneca, all outside the submitted work. In addition, JA has a patent allogenic tumor cell lysate licensed to amphera, a patent combination immunotherapy in cancer pending, and a patent biomarker for immunotherapy pending (all outside submitted work) JV reports grants from ZonMW, during the conduct of the study.

The remaining authors declare that the research was conducted in the absence of any commercial or financial relationships that could be construed as a potential conflict of interest.
